# Supplementary material for: Fast spin exchange across a multielectron mediator
Source: Nat Commun. 2019 Mar 13;10:1196. doi: 10.1038/s41467-019-09194-x (PMC6416330; doi:10.1038/s41467-019-09194-x)
Supplement: Supplementary file 1 — Supplementary Information [file 41467_2019_9194_MOESM1_ESM.pdf]

Supplementary Information for “Fast spin exchange across a multielectron mediator”

Malinowski et al.

Contents

Supplementary Note 1: Determination of gate-voltage pulses 2

Supplementary Note 2: Level structure of the multielectron dot inferred from spin-leakage spectroscopy 3

Supplementary Note 3: Extraction of joint probabilities from histograms 5

Supplementary Note 4: Measurement of the charge distribution at the spin-interaction points 6

Supplementary Note 5: Complementary fingerprint patterns obtained by detecting the spin state of the left double dot 7

Supplementary Note 6: Fingerprint pattern for an odd occupancy of the multielectron dot 7

Supplementary Note 7: Hubbard model of the exchange interaction 7

Supplementary Note 8: Effects arising from finite rise time of apparatus 10

Supplementary Note 9: Relation to spin exchange in doubly-occupied triple dots and RKKY interaction 11

References 11

Supplementary Note 1: Determination of gate-voltage pulses

The linear geometry of the quintuple quantum dot makes it difficult to measure a five-dimensional five-dot charge stability diagram: the central dot can exchange electrons with the reservoirs only via (co-)tunneling through the left or right double dot, which is strongly suppressed, particularly once the device is tuned up. Instead of mapping out full charge stability diagrams in order to determine pulse amplitudes and pulse directions in gate-voltage space, we proceed in steps. First, we choose the readout voltages V_j^{R} (which we refer to as readout point), then the separation voltages V_j^{S} (referred to as separation point), and finally interaction voltages V_j^{I} (referred to as interaction point). Here, $j \in \{\mathrm{L1}, \mathrm{L2}, \mathrm{M}, \mathrm{R1}, \mathrm{R2}\}$ (see Fig. 1a). A typical waveform cycle used to move between these points is illustrated in Supplementary Figure 1.

For each double dot (i.e. left and right double dot separately) we establish a partial charge stability diagram, by sweeping its plunger gates ($V_{\mathrm{L1,L2}}$ or $V_{\mathrm{R1,R2}}$) while monitoring its charge sensor. This can be done despite the presence of the multielectron dot, provided that plunger gates are swept sufficiently slow to allow exchange of electrons between the double dots and their reservoirs. (Compared to traditional double dots, each double dot only has one reservoir just to the left (right) of the left (right) double dot shown in Figure 1a, which may require cotunneling processes in order to transition into the charge ground state of the inner quantum dots.) Application of (unoptimized) pulse sequences (corresponding to double-dot leakage-spectroscopy measurement at fixed, finite magnetic field) allows us to optimize the static gate voltages associated with each double dot and charge sensor to obtain suitable single-shot readout performance. These readout voltages V_j^{R} (indicated with red and green in Suppl. Fig. 1) define the origin of our coordinate system. With respect to V_j^{R} we then define detuning parameters $\varepsilon_{\mathrm{L}} = (V_{\mathrm{L2}} - V_{\mathrm{L1}})/\sqrt{2}$ and $\varepsilon_{\mathrm{R}} = (V_{\mathrm{R1}} - V_{\mathrm{R2}})/\sqrt{2}$ for each double dot.

Having defined $\varepsilon_{\mathrm{L/R}}$ we repeat leakage spectroscopy, a generalization of "spin funnel" measurements[1,2]. For the case of one double dot coupled to a multielectron dot, this procedure is described in detail in Refs. 3,4. For this device, we apply leakage spectroscopy pulses to both double dots simultaneously, while varying values of ε_{L}, ε_{R}, and the applied magnetic field B. This yields data as in the left-most regions of Suppl. Fig. 3a,b. Phenomenologically, the flattening of the curved leakage feature towards increasing $\varepsilon_{\mathrm{L,R}}$ informs us about the decreasing strength of the residual exchange coupling within each double dot. This allows us to choose the separation point of each double dot V_j^{S}, by choosing $\varepsilon_{\mathrm{L,R}}$ such that the leakage feature lies between $B = 10$ and 20 mT. (In Suppl. Figure 1, the waveform segment corresponding to this configuration is shaded in yellow.) For instance, in Suppl. Fig. 3 we chose $\varepsilon_{\mathrm{L}}^{\mathrm{S}} = 13$ mV and $\varepsilon_{\mathrm{R}}^{\mathrm{S}} = 18$ mV as the separation point.

The separation point V_j^{S} in turn serves as a reference point for determining the interaction point V_j^{I} (indicated with dark blue in Suppl. Fig. 1), at which the multielectron-dot-mediated exchange interaction is induced. In case of the data presented in Figs. 2b,c and Suppl. Fig. 3a,b the interaction point V_j^{I} is parametrized by $\varepsilon_{\mathrm{M}}^{\mathrm{cc}}$ according to the formula:

$$\begin{pmatrix} V_{\mathrm{L1}}^{\mathrm{I}} \\ V_{\mathrm{L2}}^{\mathrm{I}} \\ V_{\mathrm{M}}^{\mathrm{I}} \\ V_{\mathrm{R1}}^{\mathrm{I}} \\ V_{\mathrm{R2}}^{\mathrm{I}} \end{pmatrix} = \begin{pmatrix} V_{\mathrm{L1}}^{\mathrm{S}} \\ V_{\mathrm{L2}}^{\mathrm{S}} \\ V_{\mathrm{M}}^{\mathrm{S}} \\ V_{\mathrm{R1}}^{\mathrm{S}} \\ V_{\mathrm{R2}}^{\mathrm{S}} \end{pmatrix} + \frac{\varepsilon_{\mathrm{M}}^{\mathrm{cc}}}{\sqrt{35}} \begin{pmatrix} -3 \\ -2 \\ 3 \\ -2 \\ -3 \end{pmatrix} \tag{1}$$

Equation 1 implements negative voltage pulses on plunger gates of the two double dots, with the intention of suppressing exchange of electrons between the quintuple dot and the reservoirs during the (positive) interaction pulse on V_{M}. With this choice, the "cross-compensation" pulses applied to $V_{\mathrm{L1,L2,R1,R2}}$ are *proportional* in amplitude to $\varepsilon_{\mathrm{M}}^{\mathrm{cc}}$, which distinguishes these pulses from ε_{M} pulses (which employ *constant* cross-compensation pulses, as described below). The normalization factor $\sqrt{35}$ ensures that a change of $\varepsilon_{\mathrm{M}}^{\mathrm{cc}}$ by 1 mV corresponds to a distance of 1 mV in the gate voltage space with a Cartesian metric.

We found that it is not necessary to fine tune the amplitudes of the compensating pulses, presumably due to the effective isolation of the multielectron dot from the reservoirs[5]. This simplifies the generation of subnanosecond pulses, as explained in Methods. Therefore, for some data sets we only vary $V_{\mathrm{M}}^{\mathrm{I}}$, $V_{\mathrm{L2}}^{\mathrm{I}}$, and $V_{\mathrm{R1}}^{\mathrm{I}}$. In these cases, we parametrize the interaction point V_j^{I} by parameters ε_{M} and ε:

$$\begin{pmatrix} V_{\mathrm{L1}}^{\mathrm{I}} \\ V_{\mathrm{L2}}^{\mathrm{I}} \\ V_{\mathrm{M}}^{\mathrm{I}} \\ V_{\mathrm{R1}}^{\mathrm{I}} \\ V_{\mathrm{R2}}^{\mathrm{I}} \end{pmatrix} = \begin{pmatrix} V_{\mathrm{L1}}^{\mathrm{S}} \\ V_{\mathrm{L2}}^{\mathrm{S}} \\ V_{\mathrm{M}}^{\mathrm{R}} \\ V_{\mathrm{R1}}^{\mathrm{S}} \\ V_{\mathrm{R2}}^{\mathrm{S}} \end{pmatrix} + \begin{pmatrix} 0 \\ 0 \\ \varepsilon_{\mathrm{M}} \\ 0 \\ 0 \end{pmatrix} + \frac{\varepsilon}{\sqrt{2}} \begin{pmatrix} 0 \\ -1 \\ 0 \\ +1 \\ 0 \end{pmatrix} + \frac{\varepsilon_{\mathrm{X}}}{\sqrt{35}} \begin{pmatrix} -3 \\ -2 \\ 0 \\ -2 \\ -3 \end{pmatrix}. \tag{2}$$

This parametrization enables us to fine-tune the interaction time with subnanosecond resolution by varying only ε_{M}, thereby needing only one additional channel of the arbitrary waveform generator (Methods). Physically, the parameter ε controls the relative detuning between the chemical potential of the left and right inner dot. The parameter ε_{X} (which we keep fixed) implements cross-compensation amplitudes that are independent of ε_{M}.

Aside from this technical difference between pulses parametrized by $\varepsilon_{\mathrm{M}}^{\mathrm{cc}}$ (Eq. 1) and ε_{M} (Eq. 2), data presented in this article was acquired using slightly different

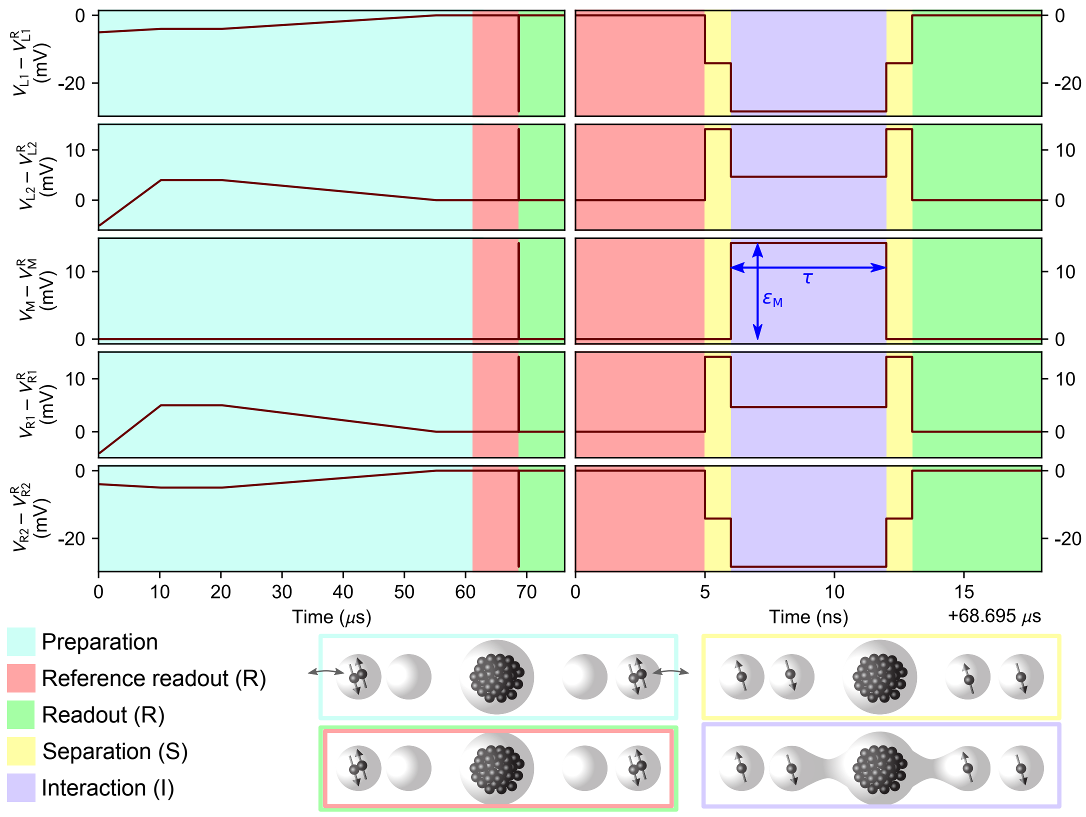

SUPPL. FIG. 1. **Detailed waveform cycle.** One typical waveform cycle applied to each of the electrostatic gates indicated in blue in Fig. 1a. Such a cycle is repeated many times to gather readout statistics (single shot histograms), and modified appropriately to determine dependences on pulse parameters $\varepsilon_{\mathrm{M}}, \tau$, etc. For clarity, the segments inducing the exchange interaction across the multielectron dot interaction are plotted using units of nanoseconds, rather then microseconds (see breaks in horizontal axes). Background colors indicate the purpose of each segment within the cycle, see legend. Cartoons in the bottom right indicate the charge configuration during each of the segments. Rise time effects (discussed in Supplementary Note 8) are ignored in this figure.

voltages applied to the static gate electrodes, as well as different choices of V_j^{S} and ε_{X} (Table 1). However, the general principle of tuning up pulse sequences in either case was similar to the procedure described here. No significant retuning of the quintuple-dot array was necessary in between different data sets, and therefore the tunnel couplings can be considered unchanged throughout the experiment, with the exception of configuration 5 and 6 (Fig. 3c,f) where tunnel coupling were intentionally reduced. However, precise choices of measurement points (V_j^{R}), separation points (V_j^{S}), sensor settings, as well as ε_{X} were adjusted between data sets.

SUPPL. TABLE 1. Static tuning configurations used in different measurements.

DC configuration	Figures
1	2a, 3b 4b, suppl. 4
2	4a
3	2b,c, suppl. 2
4	suppl. 3
5	3c
6	3d

Supplementary Note 2: Level structure of the multielectron dot inferred from spin-leakage spectroscopy

If two spin states with different total spin projection $\hat{S}_z$ are brought together in energy for a sufficiently long time, leakage from one state to the other can occur due to higher-order (non-spin-conserving) elastic processes. This provides an experimental method, spin leakage spectroscopy[4], to experimentally detect discrete states, and to quantify the exchange interaction by comparison with the Zeeman energy. In the conventional case of a double dot it can be used to locate the position of the crossing between the singlet $|S\rangle$ and the fully polarized triplet $|T_{+/-}\rangle$ state (the sign of the electronic g-factor defines which of the triplet states is used), and results in the characteristic funnel shape[1,2]. In the case of a triple quantum

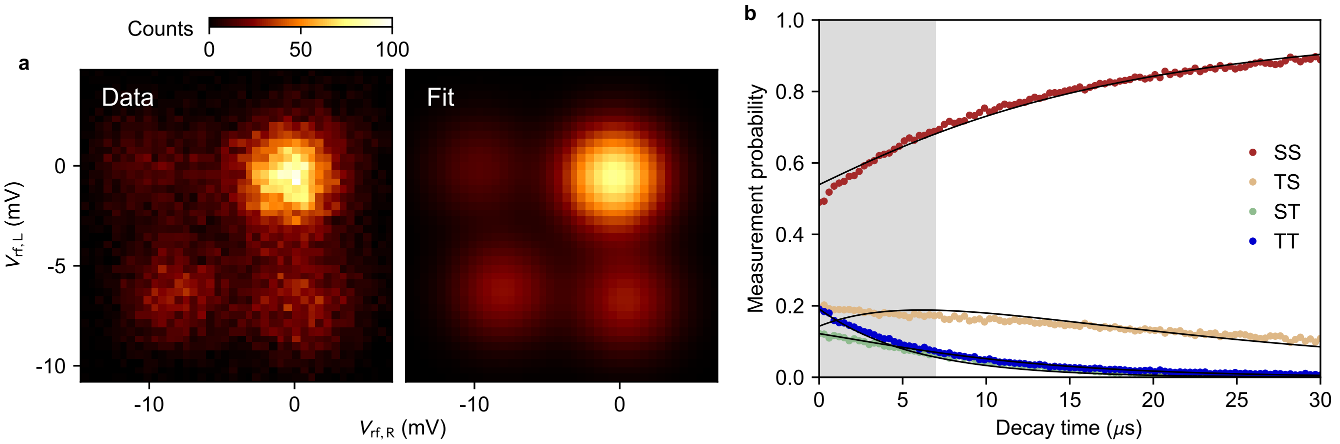

SUPPL. FIG. 2. **Probability estimation.** **a** Two-dimensional histogram of measured single-shot readouts (left) and a fitted quadrupole Gaussian (right). **b** Decay of the triplet states in the measurement configuration. The experimentally measured decay (dots) is fitted by a simple model based on two independent decay rates for the two double quantum dots (lines).

dot the position of an analogous crossing, which depends on the value of the external magnetic field, enables the reconstruction of the exchange profile[3,4,6]. Here we employ the same technique to the case of the two double quantum dots coupled to the multielectron dot.

The sequence of the applied voltage pulses is the same as the one used to detect exchange oscillations mediated by the multielectron quantum dot, except that the interaction time is increased to $\tau = 150$ ns. This time is sufficiently long to allow leakage from the initialized state into other states, for those pixels for which a level crossing occurs at the interaction point. (Moreover, 150 ns is sufficiently long to wash out any remaining coherent exchange oscillations, thanks to dephasing from Overhauser field fluctuations and charge noise.) Such pixels therefore show a suppression of P_{S}. For example, the left panels of Suppl. Fig. 3a,b present the position of the S-T_+ crossing for the two double quantum dots, acquired simultaneously. Each one can be viewed as the conventional "spin funnel" of a singlet-triplet qubit[1,2]. Note that the horizontal axes in Suppl. Fig. 3a and Suppl. Fig. 3b correspond to different gate-voltage parameters, namely detuning within the left (ε_{L}) and the right (ε_{R}) double quantum dot. Therefore the apparent similarity between the two funnels does not imply any interactions across the multielectron dot; it merely indicates that the intradot exchange coupling within the left and right double quantum dot had been tuned up with similar strenghts.

The right panels of Suppl. Fig. 3a,b present the result in the regime where indirect exchange across the multielectron dot turns on. In this part of the panels the horizontal axis is shared, and denotes the pulse amplitude $\varepsilon_{\mathrm{M}}^{\mathrm{cc}}$. We observe that for intermediate values of $\varepsilon_{\mathrm{M}}^{\mathrm{cc}}$, the leakage features detected from the left (blue dotted lines) occur at different magnetic field values compared to the leakage features detected from the right (red dotted line). The associated level crossings therefore belong to different states. In contrast, for high values of $\varepsilon_{\mathrm{M}}^{\mathrm{cc}}$, the leakage feature detected from the left and right occur at exactly the same magnetic field values (green dotted line), and diverge towards increasing field.

This agrees with a simplistic Heisenberg model of four exchange-coupled spin-1/2 dots arranged in a linear array (i.e. the multielectron dot is simply treated as a tunnel barrier). The associated energy diagram, arising from an appropriate choice of the three pairwise exchange interactions within the array, allows us to identify the observed features (Suppl. Fig. 3c). In the left side of the diagram, only exchange coupling within each double quantum dot is nonzero (and the associated spin states can be written as product states between states on the left double dot, and states on the right double dot). In the right side of Suppl. Fig. 3c, however, it is the exchange interaction mediated by the multielectron quantum dot that becomes nonzero (and associated spin states can no longer be written as product states between left and right).

The leakage features in the left part of Suppl. Fig. 3a,b correspond to the S-T_+ crossing of the left and the right double dot. In the four-dot notation, these are $|SS\rangle$-$|T_+S\rangle$ and $|SS\rangle$-$|ST_+\rangle$ crossings, which are indicated by a blue and a red triangle, respectively. In the middle part of the energy diagram and leakage spectroscopy data, $|T_+S\rangle$ and $|ST_+\rangle$ states start to hybridize due to exchange mediated by the multielectron dot. As a result, only one of the leakage features continues (the one indicated with the red-to-green dotted line), while the other leakage feature stops (blue dotted line). This indicates the position at which $|T_+S\rangle$ and $|ST_+\rangle$ are no longer eigenstates, but their superposition $(|T_+S\rangle - |ST_+\rangle)/\sqrt{2}$ is (indicated by a green line in Suppl. Fig. 3b). At this position, the $|SS\rangle$ state too is no longer an eigenstate, but instead $|\uparrow S \downarrow\rangle$ and $|\downarrow S \uparrow\rangle$ are (indicated by the orange lines). In this

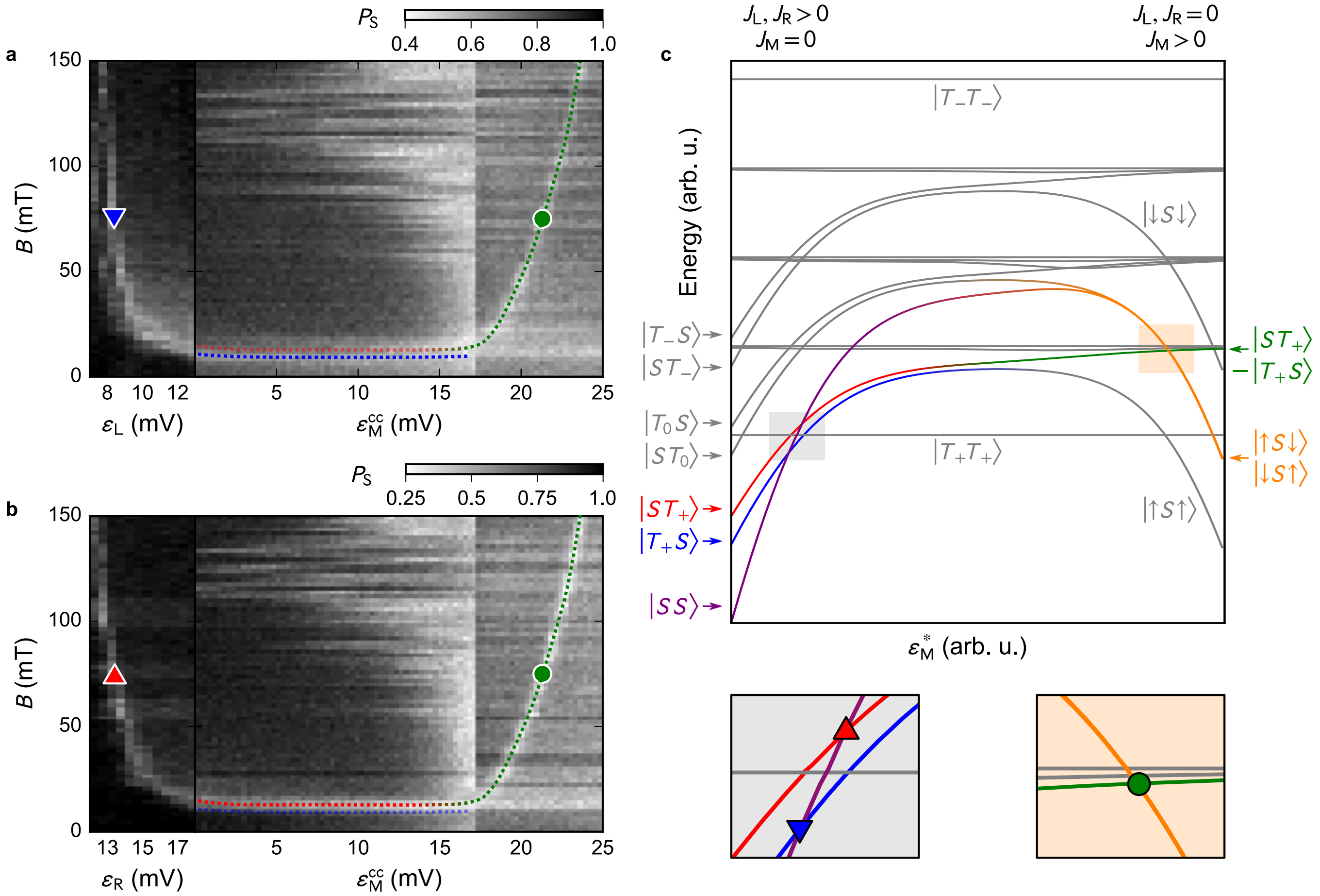

SUPPL. FIG. 3. **Leakage spectroscopy using two exchange-coupled double quantum dots.** Leakage spectroscopy measurement performed simultaneously for the left (**a**) and the right (**b**) double quantum dot. Data along ε_M^{cc} was acquired as two separate data sets with different sweep time between reference and separation points, resulting in a vertical artifact at $\varepsilon_M^{cc} = 17$ mV. The bright horizontal feature at $B \approx 15$ mT for $\varepsilon_M^{cc} > 17$ mV is a result of leakage during the separation step of the applied pulse sequence (cf. Supplementary Section) and does not indicate an additional spin-state crossing. **c** Schematic energy diagram of the two exchange-coupled double quantum dots, for finite in-plane magnetic field. In the left only the exchange interaction within the left and right double quantum dot ($J_{L/R}$) is non-zero. In the right only exchange mediated by the multielectron quantum dot (J_M) is non-zero. The multielectron dot plays the role of a barrier between the double dots (see text), and hence the kets denote the spin state of the left and right double dot only. Markers indicate the crossings detected by leakage spectroscopy measurements.

notation the left/right arrow indicates the spin state of the left/right outer quantum dot, respectively, while the symbol S indicates a spin-singlet state of the electrons on the two inner dots.

Supplementary Note 3: Extraction of joint probabilities from histograms

The joint probabilities, presented in Fig. 2b, are calculated based on histograms of single-shot outcomes of the demodulated sensor voltages for each pulse amplitude (presented in the Supplementary Video 1). For calibration purposes, we first sum multiple histograms associated with different pulse amplitudes, in order to get sufficient counts for all four outcomes SS, ST, TS, TT. To this two-dimensional histogram, we fit a two-dimensional quadruple Gaussian to obtain the position of the four peaks (8 parameters) and their widths (2 parameters, one of which sets the widths for the sensor signals of the left double quantum dot, and the other sets the widths of the sensor signals of the right double quantum dot). The data and the fit are presented in Suppl. Fig. 2a. Having fixed the positions and widths associated with all four Gaussians, we leave their amplitudes as free fit parameters when fitting histograms separately for each voltage pulse amplitude. The normalized amplitudes of the Gaussians yield the measured joint probabil-

ities $\vec{p}_{\rm meas} = (p_{\rm SS}, p_{\rm TS}, p_{\rm ST}, p_{\rm TT})$, uncorrected for charge relaxation of the two-electron double-dot states during the measurement interval.

To correct for the decay of the two-electron states in the left and right double dot during the measurement interval, we fix the amplitude of the exchange-inducing pulse at a value that yields significant number of counts for all four possible outcomes, and introduce a waiting time in the readout configuration before performing measurement of the sensor signals. This provides a measurement of the relaxation time, as exemplified in Suppl. Fig. 2b. We fit the data assuming independent relaxation rates, different for the two double quantum dots. This model yields:

$$\vec{p}(t) = M(t)\vec{p}(0) = \begin{pmatrix} 1 & 1-e^{-\Gamma_{\rm L}t} & 1-e^{-\Gamma_{\rm R}t} & 1-e^{-\Gamma_{\rm L}t}-e^{-\Gamma_{\rm R}t}+e^{-(\Gamma_{\rm L}+\Gamma_{\rm R})t} \\ 0 & e^{-\Gamma_{\rm L}t} & 0 & e^{-\Gamma_{\rm L}t}-e^{-(\Gamma_{\rm L}+\Gamma_{\rm R})t} \\ 0 & 0 & e^{-\Gamma_{\rm R}t} & e^{-\Gamma_{\rm R}t}-e^{-(\Gamma_{\rm L}+\Gamma_{\rm R})t} \\ 0 & 0 & 0 & e^{(-\Gamma_{\rm L}-\Gamma_{\rm R})t} \end{pmatrix} \vec{p}(0) \tag{3}$$

where $\Gamma_{\rm L} = 0.5$ MHz and $\Gamma_{\rm R} = 0.13$ MHz are relaxation rates in the left and right double quantum dot, respectively.

Having fitted the decay rates for both double dots (see Suppl. Fig. 2b) we can reverse the relation between measured probabilities and the actual probabilities:

$$\vec{p}_{\rm meas} = \frac{1}{T_{\rm R}} \int_0^{T_{\rm R}} M(t)\vec{p}_{\rm act}{\rm d}t \tag{4}$$

where $\vec{p}_{\rm meas/act}$ are the vectors of measured/actual outcome probabilities, $M(t)$ captures the decay during the waiting time t and $T_{\rm R}$ is the total readout time of 7 μs (as indicated with the gray-shaded region in Suppl. Fig. 2b). The integration is performed to include decay that occurs *during* the readout time. Application of the numerically inversed relation 4 yields the calculated joint probabilities of the four states, reported in Fig. 3e.

Supplementary Note 4: Measurement of the charge distribution at the spin-interaction points

In an attempt to independently confirm the position of the electrons during the interaction step, we extend the interaction time to 4 μs while keeping the remainder of the pulse sequence unchanged (the nanosecond-scale interaction times used in Fig. 3 would be too short to allow the radiofrequency tank circuits to respond). During this 4 μs-long time we apply a radiofrequency measurement tone to both charge sensors, and record their (demodulated) response while varying ε and $\varepsilon_{\rm M}$. Due to the capacitive cross-coupling between gate electrodes of the quintuple dot and the sensor quantum dots, we acquire such charge-sensing maps for several different settings of the charge sensors. This is needed because a sensor signal is sensitive to charge in the device only when the sensor dot's operating point falls on the positive or negative slope of one of its Coulomb-oscillation conductance peaks. We perform the numerical derivative of each data set along ε, then apply blur by convolving the result with a Gaussian kernel ($\sigma = 1.5$ pixel), and take the absolute value to remove sign changes of the sensor's sensitivity when its operating point switches from the positive slope to the negative slope. Finally, we sum the obtained data sets with different weights, for best visibility of charge

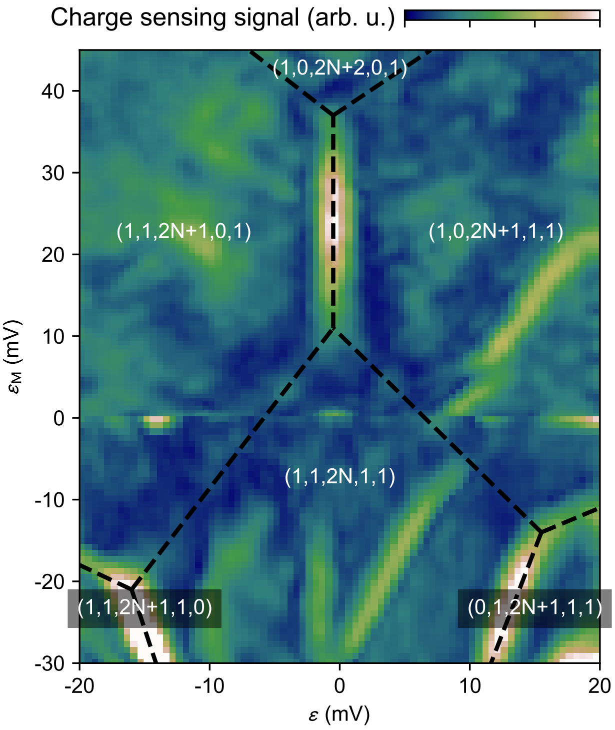

SUPPL. FIG. 4. **Charge configuration at the spin interaction point.** Processed diagram of the charge distribution during the interaction mediated by the multielectron quantum dot. Dashed lines indicate the inferred location of the charge transitions.

transitions within the device. The processed data obtained in this way is presented in Suppl. Fig. 4. Our interpretation in terms of charge transitions within the five-dot array are indicated with dashed black lines. The features corresponding to the electron transfer from each of the inner dots to the multielectron dot appear only weakly, likely due to the large tunnel coupling chosen for investigating the superexchange regime. The two additional regions in the bottom left and right of Suppl. Fig. 4 correspond to, respectively, $(1,1,2N+1,1,0)$ and $(0,1,2N+1,1,1)$ charge configurations of the quintuple quantum dot, which are partially visible also in Fig. 3b. They correspond to the relocation of the outer reference spins to the inner dots, and hence we do not inspect these regions further.

In addition to the indicated charge transitions, several other strong features are observed in Suppl. Fig. 4, which have no counterparts in the data of exchange oscillations (Fig. 3b). We associate these with artifacts arising from the long interaction time of 4 μs, which allows charge transitions within the metastable electron configuration of the quintuple dot. As long as these relaxation processes are sufficiently slow, they are irrelevant when operating exchange oscillations with short τ^5, and possibly could be suppressed by suitable cross compensation pulses.

Our interpretation of Suppl. Fig. 4 is corroborated by our simulations in Figs. 3e and 3f as well as recent work in Supplementary Reference 5, where arrays of quantum dots are operated in metastable states in an overall isolated regime.

Supplementary Note 5: Complementary fingerprint patterns obtained by detecting the spin state of the left double dot

Figures 3 and 4 presented fingerprint patterns by plotting singlet-outcomes measured for the *right* double quantum dot. Consistent with the correlations established in Fig. 2, simultaneous measurements of singlet outcomes for the *left* double dot are essentially identical, apart from a lower contrast arising from a reduced readout fidelity of the left double-dot spin state. For completeness, fingerprint patterns obtained from the left double dot are plotted in Fig. 5.

Supplementary Note 6: Fingerprint pattern for an odd occupancy of the multielectron dot

For the even-occupied multielectron quantum dot discussed above, the observed fingerprint patterns obtained from the left and right double dot are essentially identical. In striking contrast, after addition of one electron to the multielectron quantum dot the patterns detected by left and right sensor quantum dot are manifestly different, as shown in Fig. 6a and b.

Our interpretation of the observed patterns, illustrated in Fig. 6c, is as follows. In regime I both inner dots are occupied by a single electron, while the odd-occupied multielectron dot hosts a spin 1/2. Due to a significant detuning between the three dots relative to the relevant tunnel couplings, the exchange interaction neighboring spins is weak, and neither left nor right charge sensor reveals an oscillatory pattern. In regime IIa (IIb) the electron from the right (left) inner dot is transferred into the multielectron dot and exchange-interacts with the multielectron spin 1/2. This results in an oscillatory pattern in regime IIa and b detected predominantly by the right and left charge sensor, respectively. To a small degree, this oscillatory pattern is faintly visible also in the other sensor signal, likely due to a small direct capacitative coupling between one double dot and the charge sensor of the other double dot. The oscillatory pattern disappears for $\varepsilon_\mathrm{M} \gtrsim 35$ mV in regime IIa, and for $\varepsilon_\mathrm{M} \gtrsim 25$ mV in regime IIb, which we speculate to be a result of fast relaxation to the ground state of the $N+2$-occupied multielectron dot. Finally, in regime III an oscillatory fringe pattern is recovered, but it is detected predominantly by the left charge sensor for $\varepsilon \lesssim 0$ and by the right sensor for $\varepsilon \gtrsim 0$.

We speculate that in regime III the two electrons relocate *sequentially* from the inner dots into the multielectron dot. The first electron transferred into the multielectron dot interacts with the host spin-1/2 and relaxes quickly to the ground state with spin-1 (see discussion in). Subsequently, this spin-1 interacts coherently with the second electron transferred into the multielectron quantum dot, leading to the oscillatory fringe pattern. Thus, the order in which the transfer of the two electron occurs determines whether the oscillatory pattern is predominantly detected by the left or right charge sensor.

Supplementary Note 7: Hubbard model of the exchange interaction

Our simulations of exchange fringes, presented in Fig. 3e, have been obtained using a phenomenological model for a multielectron quantum dot outlined in Ref. 4 (see also Ref. 7), by adding terms which describe two tunnel-coupled single-electron quantum dots. For simplicity we only model the three dots in the center of the quintuple-dot array. The outer dots are decoupled from the inner dots for the period of interaction. Therefore, they will not contribute to the effective exchange mediated by the middle dot and are ignored in the model. (Also, while most experimental control parameters are voltages, such as $\varepsilon, \varepsilon_\mathrm{M}$ etc, corresponding parameters in our model are energies, such as $\varepsilon^*, \varepsilon^*_\mathrm{M}$ etc. Because of the negative electronic charges, an increase in ε_M in the experiment, for instance, corresponds in the simulation to making ε^*_M more negative.) Since the multielectron dot has a spinless ground state, we neglect the electron pairs that are singlet-paired in the orbitals below the Fermi

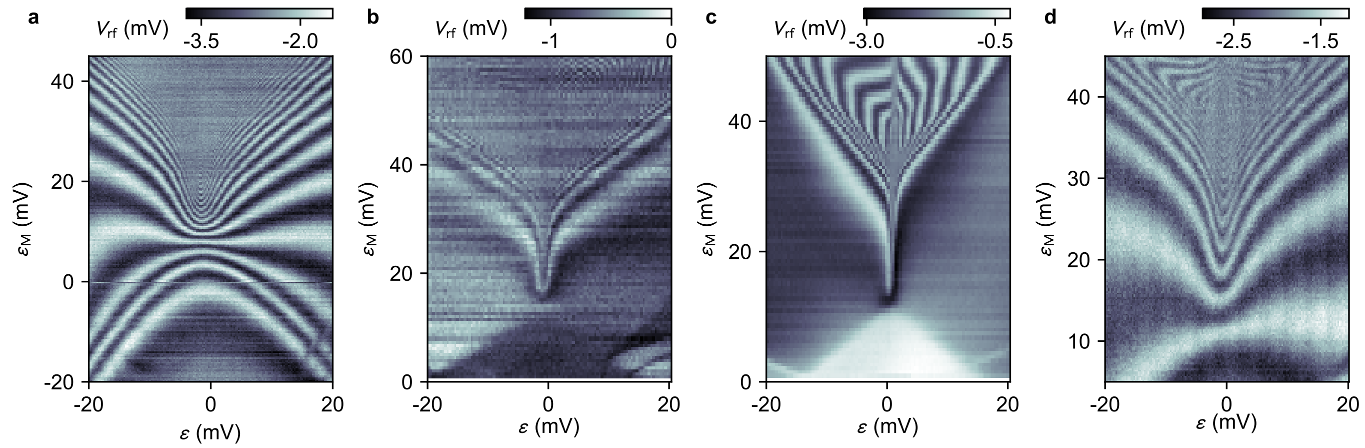

SUPPL. FIG. 5. **Fingerprint patterns obtained from the left double dot.** Plotting singlet outcomes measured for the left double dot reveals fingerprint patterns that are essentially identical to the ones observed for the right double dot. The panels **a,b,c,d** present data sets complementary to panels 3b, 3c, 3d and 4a, respectively.

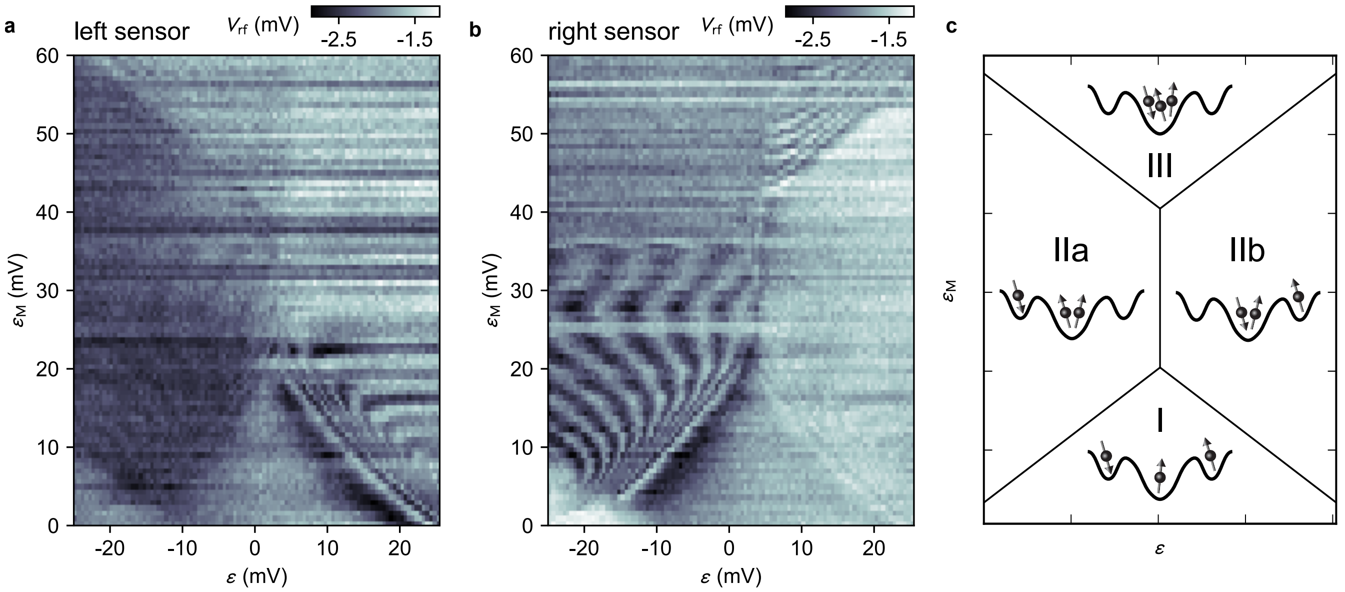

SUPPL. FIG. 6. **Fingerprint patterns for odd occupancy of the multielectron quantum dot. a,b** Simultaneously measured fingerprint pattern for $\tau = 4$ ns as detected by left and right charge sensor. **c** Illustration of the electron configuration in different regions observed in **a,b**. The effective spin 1/2 located in the multielectron quantum dot is represented by the same symbol as single electrons.

energy. We also neglect all but the two lowest unoccupied orbitals, such that the three dots are described by a Hubbard model with four orbitals. The labels L and R denote the orbitals of the two inner dots, and labels 1 and 2 correspond to the two, non-degenerate orbitals of the middle dot. The Hubbard Hamiltonian of the system (illustrated in Fig. 7) is given by

$$\hat{H} = \sum_i \left(\varepsilon_i^* \hat{n}_i + \frac{U_i}{2} \hat{n}_i (\hat{n}_i - 1) \right) + \sum_{i \neq j} \frac{K_{ij}}{2} \hat{n}_i \hat{n}_j + \\ - \frac{\xi}{2} \hat{S}^2 - \sum_{\langle i,j \rangle} \sum_\alpha t_{ij} (c_{i,\alpha}^\dagger c_{j,\alpha} + \text{H.c.}), \quad (5)$$

which sums over the orbitals $i = L, 1, 2, R$ and electron spin orientations $\alpha = \uparrow, \downarrow$. The operator $\hat{n}_i = \sum_\alpha c_{i,\alpha}^\dagger c_{i,\alpha}$ counts the number of electrons in orbital i. As shown in Fig. 7, ε_i^* describes the gate-tunable chemical potential

of each orbital. U_i and K_{ij} capture, respectively, intra- and inter-orbital Coulomb interaction energies. The term proportional to ξ describes the spin correlation energy of the middle dot, favoring $S = 1$ triplet configurations when both orbitals 1 and 2 are occupied. $\hat{S}$ is the total spin operator for the middle dot where spin in each orientation ℓ is given by $\hat{S}^{\ell} = \frac{1}{2}\sum_{\lambda,\alpha,\alpha'} c^{\dagger}_{\lambda,\alpha}\sigma_{\alpha,\alpha'}c_{\lambda,\alpha'}$, summed over the orbitals $\lambda = 1, 2$. The final term in the Hamiltonian denotes tunnel couplings t_{ij} between orbitals $\langle i, j\rangle$ located in adjacent dots.

We implement specific multi-dot voltage pulses to explore the regimes of the effective exchange interaction. Therfore, we rewrite the orbital parameters (shown in Fig. 7) as

$$\varepsilon_{\mathrm{S}} = \varepsilon_2^* - \varepsilon_1^*, \quad \bar{\varepsilon} = \frac{\varepsilon_{\mathrm{L}}^* + \varepsilon_1^* + \varepsilon_{\mathrm{R}}^*}{3},$$
$$\varepsilon^* = \frac{\varepsilon_{\mathrm{L}}^* - \varepsilon_{\mathrm{R}}^*}{2} \quad \varepsilon_{\mathrm{M}}^* = \varepsilon_1^* - \frac{\varepsilon_{\mathrm{L}}^* + \varepsilon_{\mathrm{R}}^*}{2}. \quad (6)$$

The first term, ε_{S}, is the spacing between the first and second orbitals of the middle dot. In our model, ε_{S} is determined by the mesoscopic details of the dot and is independent of the plunger gate voltage V_{M}, so we take this as a fixed parameter. Tuning the second term, $\bar{\varepsilon}$, while keeping all others in Eqn. 6 constant is equivalent to a uniform voltage pulse on all dots, so we neglect it. The next term, ε^*, sets the difference between the chemical potentials of the left and right dots. This is proportional to the gate voltage ε, up to some lever arm factor, in addition to a factor of $1/\sqrt{2}$ arising from a difference in definition. The last term, $\varepsilon_{\mathrm{M}}^*$, controls the detuning of the middle dot chemical potential relative to the left and right dots. This is proportional to the gate voltage V_{M} again up to some lever arm. We have reduced the four gate-tunable chemical potentials ε_i in our Hubbard model to two variables, ε^* and $\varepsilon_{\mathrm{M}}^*$, which will affect exchange. These terms are, respectively, the x and y axes of Fig. 3e and 8.

There are numerous parameters in the Hubbard model whose values need to be fixed to calculate exchange oscillations for Fig. 3e. However, exact values for most terms, particularly the various Coulomb energies, U_i and K_{ij}, are not known. Fortunately, the charging energy of the multielectron dot is known to be approximately 1 meV. Therefore, we employ some simplifying assumptions to reduce the number of parameters in the model, summarized in Table 2. We expect the middle-dot intra-orbital Coulomb interaction energies U_1 and U_2 and the inter-orbital Coulomb interaction energy K_{12} to be comparable, so we assume that they are equal. This is convenient, as it allows us to define an energy scale U, approximately equal to the 1 meV charging energy of the mid-dle dot and proceed by defining the remaining Coulomb terms relative to U. The left and right dots are smaller than the middle dot, and are expected to have an appropriately larger intra-orbital Coulomb interaction energy. Based on their size relative to the middle dot, we denote $U_{\mathrm{L}} = U_{\mathrm{R}} \approx 5U$. The remaining K_{ij} terms have been estimated based on the spacing between dots in the device; nearest-neighbor terms are assumed equal and $0.10\,U$, while the next-nearest neighbor term K_{LR} is $0.02\,U$. We have set the spin correlation energy ξ based on experiments in Ref. 4 conducted on the device on the same chip. Since the Coulomb energies of the left and right dots are large with respect to the middle dot, our model will only consider tunneling events *onto* the middle dot. Furthermore, we shall assume that tunneling terms from the left and right dots to the same orbital are equal, such that the model reduces to two tunneling parameters: t_1 and t_2. The last three terms in Table 2 (ε_{S}, t_1 and t_2) represent tuning parameters for the exchange mediated by the middle dot whose values have been chosen to reproduce an oscillation pattern qualitatively similar those in Fig. 3b.

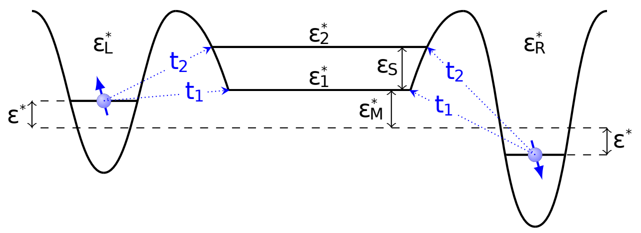

SUPPL. FIG. 7. **Schematic of a spinless multielectron dot (center) tunnel-coupled to two single-electron quantum dots (left and right).** Symbols $\varepsilon_{\mathrm{L/1/2/R}}$ label the single-particle energies of the orbitals in single-electron dots, and the two lowest unoccupied orbitals in the multielectron dot. The energy difference between the two orbitals in the multielectron dot is denoted by $\varepsilon_{\mathrm{S}} = \varepsilon_2 - \varepsilon_1$. The parameters ε^* and $\varepsilon_{\mathrm{M}}^*$ are varied to obtain Fig. 3e. Electrons in the middle dot (light blue) are singlet-paired below the Fermi energy, and are ignored in the Hubbard model. The electrons in left and right dots (blue) are only tunnel-coupled to unoccupied orbitals (blue dotted arrows). We assume that tunneling rates from left and right dots are equal.

The Hamiltonian in Eqn. 5 can be solved in the two-electron regime to extract the gate-dependent effective exchange splitting $J_{\mathrm{eff}}(\varepsilon^*, \varepsilon_{\mathrm{M}}^*)$ between the singlet and triplet states of the two spins. When the middle dot is far detuned from the left and right dots there is no exchange and the eigenstates of the Hamiltonian are $|\uparrow\downarrow\rangle$ and $|\downarrow\uparrow\rangle$. A mediated exchange interaction is induced by applying gate voltages affecting ε^* and $\varepsilon_{\mathrm{M}}^*$, resulting in flip-flops between the two electronic spins. In the simulations, we track oscillations between $|\uparrow\downarrow\rangle$ and $|\downarrow\uparrow\rangle$ by detecting the spin states of both double quantum dots via spin-to-charge conversion. This is different from the experiment where the precession occurs between $|S_{\mathrm{L}}\rangle\,|S_{\mathrm{R}}\rangle$ and $\frac{1}{2}(|S^{\mathrm{L}}\rangle\,|S^{\mathrm{R}}\rangle - |T_0^{\mathrm{L}}\rangle\,|T_0^{\mathrm{R}}\rangle + |T_+^{\mathrm{L}}\rangle\,|T_-^{\mathrm{R}}\rangle + |T_-^{\mathrm{L}}\rangle\,|T_+^{\mathrm{R}}\rangle)$ states. However, the observed pattern of oscillations is the same, except for the visibility (100 % visibility in the simu-

SUPPL. TABLE 2. Summary of the values of parameters for the Hubbard Hamiltonian for Fig. 3e. The intra-dot Coulomb interaction energy U has been used to define an energy scale to estimate the remaining parameters.

Parameter	Value (relative to U)
$U_1 = U_2 = K_{12} \equiv U$	1.00
$U_\mathrm{L} = U_\mathrm{R}$	5.00
$K_\mathrm{L1} = K_\mathrm{L2} = K_\mathrm{1R} = K_\mathrm{2R}$	0.10
K_LR	0.02
ξ	0.10
ε_S	0.06
$t_\mathrm{L1} = t_\mathrm{1R} \equiv t_1$	0.04
$t_\mathrm{L2} = t_\mathrm{2R} \equiv t_2$	0.01

lations and expected 75% visibility in the experiment), which is adjusted manually in the presented simulations. The oscillations in Fig. 3e, calculated from $J_\mathrm{eff}(\varepsilon^*, \varepsilon^*_\mathrm{M})$, describe the probability of recovering an initial state $|\uparrow\downarrow\rangle$ after an evolution time $\tau = 6$ ns, for a set of gate voltages $(\varepsilon^*, \varepsilon^*_\mathrm{M})$. The range of the ε^* and ε^*_M axes have been chosen to cover electronic configurations (1,2N,1), (0,2N+1,1), (1,2N+1,0) and (0,2N+2,0), as in Fig. 3e. Recall that the 'unoccupied' state of the middle dot describes an effective vacuum with $2N$ electrons. The evolution time τ for the Hamiltonian has been estimated using the approximation $U \approx 1$meV. Simulations in Fig. 3e qualitatively reproduce the three regimes of exchange interaction observed in Fig. 3b.

In (0,2N+2,0), the middle dot has two excess electrons, one from each adjacent dot. There are three possible two-electron states for the middle dot. There may be two electrons singlet-paired in the lowest orbital, or one electron in each orbital, forming either a triplet or singlet state. Neglecting Coulomb interactions, the latter two states are gapped from the former by ε_S when $\xi = 0$. However, the presence of the non-zero spin correlation term in Eqn. 5 lowers the energy of the triplet state by ξ. We have tuned $\varepsilon_\mathrm{S} < \xi$ such that the two-electron ground state is a triplet spin configuration. Thus, in this region the two electrons, located in the same dot, have an 'onsite' exchange splitting $J_\mathrm{eff} \approx \varepsilon_\mathrm{S} - \xi$, which is negative (triplet-favoring) and small, producing rapid oscillations.

For (0,2N+1,1)/(1,2N+1,0) the middle dot has an excess electron from the left/right dot. The electrons are now located in adjacent sites, so 'direct' exchange interaction arises from virtual occupation of the middle dot. A perturbative analysis demonstrates the exchange splitting will have the generic form $J_\mathrm{eff} \approx 2t_1^2/\Delta_\mathrm{S} - t_2^2/\Delta_\mathrm{T}$. The symbols $t_{1/2}$ are the tunneling terms from earlier, while $\Delta_\mathrm{S/T}$ describe the energy costs of an electron tunneling into either the first orbital and forming a singlet state (Δ_S), or tunneling into the second orbital and forming a triplet state (Δ_T). These Δ terms are linear combinations of gate voltages from Eqn. 6, Coulomb interaction energies and the spin correlation energy. Note that J_eff may be positive (singlet-favoring) or negative (triplet-favoring) depending on the choice of parameters. Following the previous section, since $U_1 = K_{12}$ and $\varepsilon_\mathrm{S} < \xi$, we expect $\Delta_\mathrm{T} < \Delta_\mathrm{S}$. However, when the middle dot is far detuned from the left and right dots, $\Delta_\mathrm{T} \approx \Delta_\mathrm{S}$ and the overall sign of J_eff is determined by the ratio of the tunneling terms. We set $t_1 > t_2/\sqrt{2}$ such that, in this detuned regime, J_eff is positive (singlet-favoring), opposite to the previous region. This splitting grows more positive as we tune towards (0,2N+2,0), producing more rapid oscillations. Critically, near the charge transition between (1,2N+1,0)/(0,2N+1,1) and (0,2N+2,0), we see a maximum in the exchange profile $J_\mathrm{eff}(\varepsilon^*, \varepsilon^*_\mathrm{M})$ as the dominant source of exchange changes from singlet-favoring to triplet-favoring. This maximum produces chevrons in the oscillation pattern. In Fig. 3e charge noise on the gates results in blurring that is larger than the fringe separation, obscuring the chevrons. However, they are evident in Fig. 4c, evaluated at a shorter evolution time.

Finally, in (1,2N,1), the electrons occupy distant dots and the exchange splitting approaches zero. However, there is still a weak interaction which arises from virtual occupation of the middle dot. Again, the sign of this exchange is positive since we have set $t_1 > t_2/\sqrt{2}$. However, the process involves co-tunneling events from the left and right dots. Therefore $J_\mathrm{eff} \propto t_1^4$ and the splitting is much smaller, eventually vanishing as the middle-dot orbitals are detuned even further. The oscillations in this region are correspondingly slower and eventually vanish.

Supplementary Note 8: Effects arising from finite rise time of apparatus

Due to the high frequency of the exchange oscillations and the short duration of the exchange pulses, the rise time of our apparatus has pronounced distorting effects on the observed oscillation patterns. A simulation based on square pulses would not be realistic, and hence we assume a simple phenomenological model for the time dependence of ε^*_M used in our simulations:

$$\varepsilon^*_\mathrm{M}(t) = \tilde{\varepsilon}^*_\mathrm{M} - (1.5 + \tilde{\varepsilon}^*_\mathrm{M} e^{-t/\tau_0}) \tag{7}$$

where $\tau_0 = 0.8$ ns, $\tilde{\varepsilon}^*_\mathrm{M}$ is the value displayed on the vertical axis in the figures presenting the simulations and $\varepsilon^*_\mathrm{M}(0) = -1.5$ is chosen for convenience, but its precise value has no qualitative influence on the obtained oscillations pattern.

We expect that finite-rise-time effects are most pronounced in the ε-ε_M fringe pattern obtained with short interaction time $\tau = 2$ ns, presented in Fig. 4a. Our simple model in Eq. 7 captures most, but not all distortion effects observed in this data. For example, Fig. 8 shows that a finite rise time can result in the upward bending of fringes around the symmetry point in the onsite exchange interaction regime, similar to what is observed in

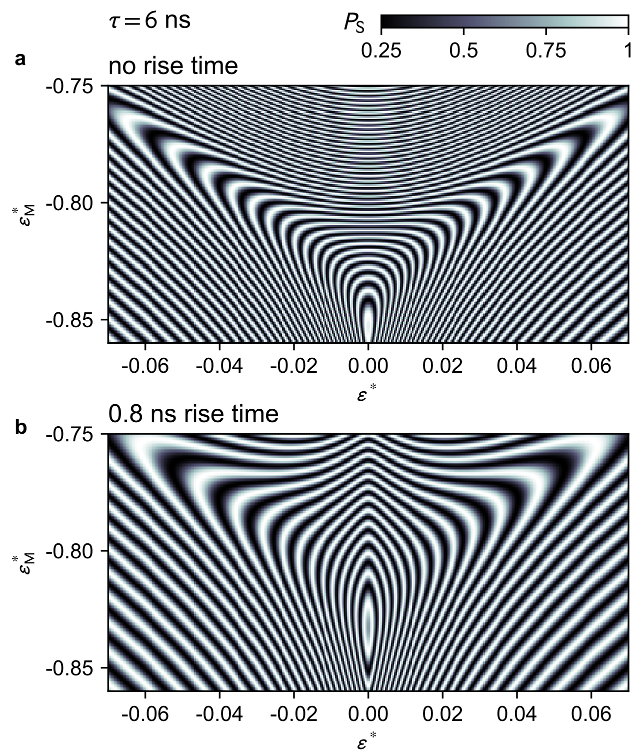

SUPPL. FIG. 8. **Effects of finite rise time.** Comparison of the simulated fingerprint at the crossover between direct and onsite exchange regimes, excluding (a) and including (b) effects of a finite rise time.

Fig. 4a. This effect can also be understood intuitively, by noting that the smaller $|\varepsilon|$ is, the larger is the exchange interaction J while V_M is rising. However, some differences between simulations and experiment remain, and may require gate-voltage-dependent parameters in the Hamiltonian of Eq. 6 and a more realistic waveform in Eq. 7.

Supplementary Note 9: Relation to spin exchange in doubly-occupied triple dots and RKKY interaction

For applications in spin-based quantum computation, it is instructive to compare the characteristics of our device, namely two singlet-triplet qubits spaced by a large multielectron dot, with recent results obtained from two closely-spaced one-electron dots with an empty one-electron dot in-between. In that triple dot experiment, spin exchange between two electrons was probed in an asymmetric charge configuration: At the electrostatic degeneracy between (2,0,0) and (1,0,1), exchange oscillations were only observed when sufficiently lowering the (1,1,0) charge state (see 8 for notation and corresponding data). Based on simulations, oscillations just before the onset of this conventional direct exchange were interpreted as "superexchange", but neither the negative sign of the claimed coupling nor its characteristic dependence on tunnel coupling strengths were observable. Phenomenologically, the most striking difference of our experiment is the observation of indirect exchange oscillations in a charge configuration that is left-right symmetric. Along with its considerate speed, such a symmetric gate may have advantages in larger qubit arrays. Our results do not directly reveal to what extent Ruderman-Kittel-Kasuya-Yosida (RKKY) interactions are involved. An experimental test of RKKY-like processes may be possible by studying increasingly larger dots, but requires a careful quantification of the distance-dependent charging energy. A quantification of tunnel matrix elements would also allow to test the fourth-order dependence on tunneling coupling in the on state (which is not unique to RKKY), and the charging-energy dependence in the off state of the interaction[9].

[1] Petta, J. R. *et al.* Coherent Manipulation of Coupled Electron Spins in Semiconductor Quantum Dots. *Science* **309**, 2180–2184 (2005).

[2] Maune, B. M. *et al.* Coherent singlet-triplet oscillations in a silicon-based double quantum dot. *Nature* **481**, 344–347 (2012).

[3] Martins, F. *et al.* Negative Spin Exchange in a Multielectron Quantum Dot. *Physical Review Letters* **119**, 227701 (2017).

[4] Malinowski, F. K. *et al.* Spin of a Multielectron Quantum Dot and Its Interaction with a Neighboring Electron. *Physical Review X* **8**, 011045 (2018).

[5] Flentje, H. *et al.* Coherent long-distance displacement of individual electron spins. *Nature Communications* **8**, 501 (2017).

[6] Gaudreau, L. *et al.* Coherent control of three-spin states in a triple quantum dot. *Nature Physics* **8**, 54–58 (2011).

[7] Deng, K., Mayhall, N. J. & Barnes, E. Negative exchange interactions in coupled few-electron quantum dots. *arXiv* arXiv: 1712.05795 (2017).

[8] Baart, T. A., Fujita, T., Reichl, C., Wegscheider, W. & Vandersypen, L. M. K. Coherent spin-exchange via a quantum mediator. *Nature Nanotechnology* **12**, 26–30 (2016).

[9] Srinivasa, V., Xu, H. & Taylor, J. Tunable Spin-Qubit Coupling Mediated by a Multielectron Quantum Dot. *Physical Review Letters* **114**, 226803 (2015).
