# Supplementary material for: Fast spin exchange across a multielectron mediator
Source: Nat Commun. 2019 Mar 13;10:1196. doi: 10.1038/s41467-019-09194-x (PMC6416330; doi:10.1038/s41467-019-09194-x)
Supplement: Supplementary file 3 — Description of Additional Supplementary Files [file 41467_2019_9194_MOESM3_ESM.pdf]

Description of Additional Supplementary Files

File Name: Supplementary Movie 1
Description: Readout histograms are presented (as in Fig. 2b) as a function of increasing amplitude of the spin exchange pulse.
